# Sonographic B-Lines, Fluid Resuscitation, and Hypoxemia in Malawian Patients with Suspected Sepsis

**DOI:** 10.1164/rccm.202003-0640LE

**Published:** 2020-08-01

**Authors:** Richard J. Wang, Grace Katha, Miriam Phiri, Philip Delbridge, Stephen B. Gordon, Carolyn S. Calfee, Laurence Huang, Jamie Rylance

**Affiliations:** ^1^ University of California San Francisco San Francisco, California; ^2^ Queen Elizabeth Central Hospital Blantyre, Malawi; ^3^ Malawi Liverpool Wellcome Clinical Research Programme Blantyre, Malawi; ^4^ Royal Liverpool University Hospital Liverpool, United Kingdom and; ^5^ Liverpool School of Tropical Medicine Liverpool, United Kingdom

*To the Editor*:

The optimal approach to fluid resuscitation for patients with sepsis is uncertain. Data from sub-Saharan Africa have indicated potential for harm from aggressive treatment with intravenous fluids (1, 2). Nevertheless, intravenous fluids remain an important therapy, and strategies to optimize their use are urgently needed.

The role of lung ultrasonography for guiding fluid management for patients with sepsis is undefined. Sonographic B-lines correlate with measurements of extravascular lung water (3–5). Increased extravascular lung water is associated with increased mortality in sepsis (6–8). Lung ultrasonography has therefore been proposed as a tool for assessing fluid tolerance in sepsis.

We recruited a cohort of 70 patients who presented to the Queen Elizabeth Central Hospital in Blantyre, Malawi, with suspected sepsis. The aims of the study were to estimate the effect of intravenous fluid treatment on lung ultrasound findings and to examine the relationship between lung ultrasound findings and the development of hypoxemia. Some results were reported previously in an abstract (9).

## Methods

Participants were a convenience sample of adults over 18 years old who presented to the Queen Elizabeth Central Hospital with suspected sepsis during weekdays, recruited between 7 a.m. and 4 p.m. Suspected sepsis was defined as an abnormal temperature measurement (≥38.0°C or <36.0°C) or symptoms of fevers, chills, or rigors in the preceding week, and evidence of hemodynamic instability (any one of the following: tachycardia >120 beats/min; systolic blood pressure <90 mm Hg; or mean arterial pressure <65 mm Hg). Written informed consent was provided by participants or their surrogates. Prisoners were excluded, as were any patients who had received more than 2 L intravenous fluid prior to screening. Ethical approval was granted by the Malawi College of Medicine (P.04/18/2385) and by the University of California San Francisco (227029).

Eight-zone lung ultrasonography was performed by an experienced operator (R.J.W. or P.D.) using a Philips S4-1 Lumify phased array transducer at enrollment, and again at 3, 6, 24, 48, and 72 hours. While participants were supine, 4-second video clips were recorded at each quadrant of the hemithorax for a total of eight clips per exam. Clips were reviewed offline by an experienced reader (R.J.W.) at a later time, and a B-line score was generated for each exam. B-lines are hyperechoic artifacts that extend from the pleura through the depth of the image. The maximal number of B-lines visualized in a single frame for each clip was summed to generate a B-line score (10).

Vital signs, including pulse oximetry on room air, were measured at each time point. The volume of intravenous fluid given between each time point was recorded. Clinical decisions regarding intravenous fluids were made by each participant’s clinical team strictly without input from research personnel.

To account for repeated measures, mixed-effects regression was used to model the relationship between the volume of intravenous fluid treatment and the B-line score at any time point, and between the B-line score and oxygen saturation. To test whether the baseline B-line score predicted impending hypoxemia, analysis was restricted to the subset of participants who were normoxemic—had an oxygen saturation of 90% or greater while breathing room air—at baseline. Simple logistic regression was used to model the baseline B-line score as the predictor and the development of hypoxemia—any measurement of oxygen saturation <90% while breathing room air—in the first 72 hours of hospitalization as the outcome. To assess the performance of the baseline B-line score for predicting impending hypoxemia, the area under the receiver operating curve was calculated, with 1,000 bootstrap replications to obtain bias-corrected 95% confidence intervals (CIs).

## Results

Clinical characteristics for the 70 participants enrolled in the study from July 18, 2018, through October 26, 2018, are shown in Table 1.

**Table 1.** Participant Characteristics

Variable			*n* (*%*) or Median (IQR) (*n* = *70*)
Physiologic variables			
Sex, F			43 (61.4)
Age, yr			35 (26–47)
Heart rate, beats per min			130 (121–142)
Systolic blood pressure, mm Hg			99 (86–115)
Systolic blood pressure ≤90 mm Hg			24 (34.3)
Mean arterial pressure ≤65 mm Hg			15 (21.4)
Respiratory rate, breaths per min			28 (22–36)
Oxygen saturation while breathing room air, %			92 (86–96)
Oxygen saturation <90% while breathing room air			30 (42.8)
Whole blood lactate, mmol/L			3.9 (2.6–7.2)
HIV-related variables			
Unknown HIV serostatus at enrollment			2 (2.9)
Negative HIV serostatus tested within 6 mo			20 (28.6)
Known positive HIV serostatus			48 (68.6)
Time since HIV diagnosis, d (*n* =40)			537 (22–1,972)
On antiretroviral therapy at enrollment			41/48 (85.4)
Sepsis treatment variables			
Cumulative amount of intravenous fluid given, ml			
Prior to enrollment			0 (0–1,000)
By 3 h after enrollment			1,000 (200–1,450)
By 6 h after enrollment			1,000 (500–2,000)
By 24 h after enrollment			1,150 (1,000–2,000)
By 48 h after enrollment			1,900 (1,000–2,750)
By 72 h after enrollment			2,000 (1,000–3,000)
Cumulative number of participants who received empiric antibiotics (ceftriaxone, 2 g intravenous)			
Prior to enrollment			21 (30.0)
By 3 h after enrollment			47 (67.1)
By 6 h after enrollment			60/66 (90.9)
By 24 h after enrollment			65/66 (98.5)
Number of participants treated with mechanical ventilation			0 (0)
Number of participants treated with a vasopressor infusion			1 (1.4)
Baseline sonographic B-line score*			5 (2–8)
In-hospital mortality			17 (24.3)

*Definition of abbreviation*: IQR = interquartile range.

* The sum of the number of B-lines observed in each of eight thoracic zones.

The volume of intravenous fluid treatment was significantly associated with the number of B-lines detected. Within the first 72 hours of hospitalization, every additional liter of intravenous fluid treatment was associated with approximately one additional B-line (1.01; 95% CI, 0.59–1.43 *P* < 0.001).

The number of B-lines detected was significantly associated with oxygen saturation. For every additional B-line detected, a participant’s oxygen saturation was decreased by 0.24% (95% CI, 0.07–0.41; *P* = 0.005).

**Figure 1.**
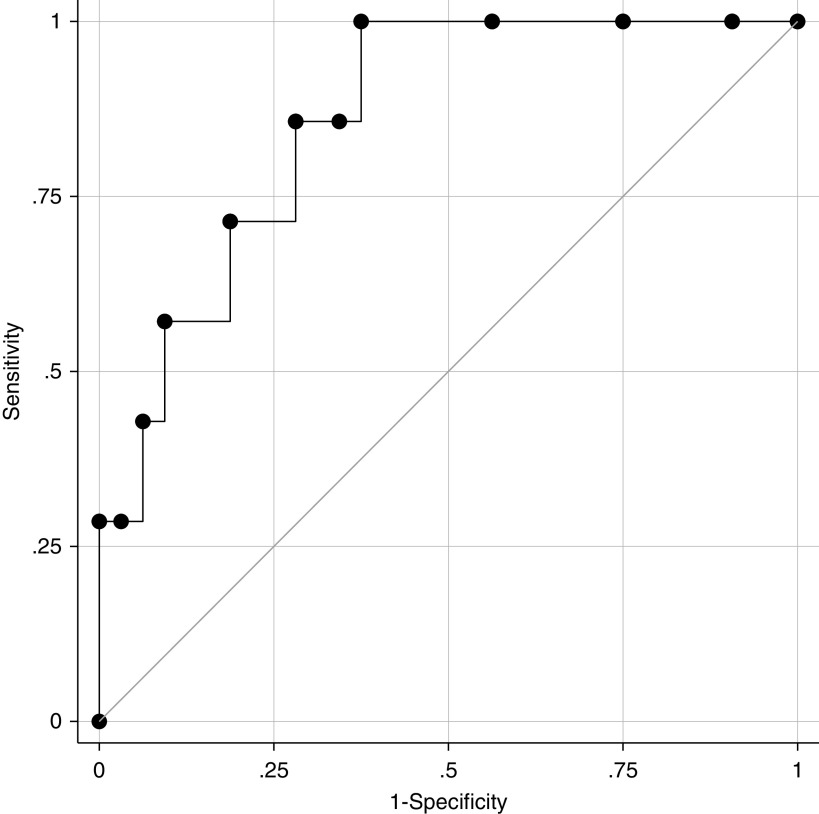
A receiver operating characteristic curve illustrating how the sonographic B-line score predicts future hypoxemia. The area under the receiver operating characteristic curve is 0.86 (95% confidence interval, 0.69–0.97).

Among the 39 participants who were normoxemic on presentation, the baseline B-line score predicted the development of hypoxemia within 72 hours of enrollment. Every additional B-line detected at baseline was associated with 1.5 (95% CI, 1.1–2.0; *P* = 0.01) times the odds of developing hypoxemia within the subsequent 72 hours. The area under the receiver operating characteristic curve (Figure 1) was 0.86 (95% CI, 0.69–0.97). A B-line score >5 was 86% sensitive and 72% specific for identifying a patient who, though normoxemic at the time of baseline examination, would develop hypoxemia within 72 hours.

## Discussion

In this study, we found a significant association between the volume of intravenous fluid received and the number of B-lines subsequently detected with lung ultrasonography. The number of B-lines detected is significantly associated with a concurrent measurement of oxygen saturation. Lastly, the baseline lung ultrasound exam might identify normoxemic patients who are at increased risk of developing hypoxemia within the first 72 hours of hospitalization. Because this is an observational study, these findings suggest, but do not establish, a potential role for using lung ultrasonography to assess fluid tolerance in patients with sepsis.

An important strength of this study is that it was performed in a hospital in Malawi, where there is a paucity of—and, perhaps, the greatest need for—data on fluid resuscitation in patients with suspected sepsis. A limitation of this study is the small sample size. A further limitation of this study is that the ultrasound exams were scored by a single experienced reader who had access to the full data set and, thus, was not formally blinded to data on intravenous fluid volume or oxygen saturation.

Despite these limitations, we believe the results of this study attest to the relationship between intravenous fluid treatment, sonographic B-lines, and clinical outcomes, such as impaired oxygenation. Testing an ultrasound-guided fluid resuscitation strategy in sepsis warrants further investigation.
